# Dose–Effect Relationship of Water Salinity Levels on Osmotic Regulators, Nutrient Uptake, and Growth of Transplanting Vetiver [*Vetiveria zizanioides* (L.) Nash]

**DOI:** 10.3390/plants10030562

**Published:** 2021-03-16

**Authors:** Jing Su, Yanhua Qiu, Xiaosong Yang, Songyan Li, Zhengyi Hu

**Affiliations:** 1 College of Resources and Environment, University of Chinese Academy of Sciences, Beijing 101408, China; sujing-418@163.com (J.S.); qiu_yan_hua@163.com (Y.Q.); yangxiaosong15@mails.ucas.ac.cn (X.Y.); lisongyan17@mails.ucas.edu.cn (S.L.); 2 Sino-Danish Center for Education and Research, Beijing 100190, China

**Keywords:** vetiver, osmotic regulators, salt tolerance, dosage effect, nutrient uptake

## Abstract

Vetiver grass [*Vetiveria zizanioides* (L.) Nash] without seeds, suitable for growing on coastal saline land, has attracted attention because of oil extraction from its roots and industrial and agricultural use. In this study, a pot experiment with different NaCl contents was used to investigate the influence of water salinity levels on vetiver, salt tolerance, and the feasibility of transferring it to coastal saline regions. The results indicated that the fresh weight of roots and shoots increased initially and then gradually decreased with an increase in NaCl content, and the maximum was attributed to a water salinity of 0.3%. The vetiver can tolerate a maximum saline content of up to 2%. The promotion of vetiver growth under water salinity could be attributed to the acceleration of nutrient uptake-induced saline, including K, N, and Cl. The growth of vetiver was insignificantly inhibited with 0.5% water salinity (mild stress), significantly inhibited with 1.0% water salinity (moderate stress: biomass decrease), and severe inhibited with >1.5% water salinity (intense stress: biomass decrease). The salt tolerance of vetiver was due to osmotic regulation by reducing sugars under mild stress and of proline under intense stress, and Na^+^ sequestration in roots and the transformation of Cl^−^ away from sensitive roots. The vetiver could be cultivated in slightly coastal saline soil (0.1–0.2% soil salinity) and even moderately saline coastal soil (0.2–0.4% soil salinity) under irrigation with low salt water during transplanting.

## 1. Introduction

There are 2270 km^2^ of coastal saline soil in north China [1,2], and most of it is low-yield fields and wastelands. Among the coastal saline soils, the Yellow River Delta, one of the three largest river deltas in China, accounts for a large proportion. High soil salinity is the main limiting factor influencing plant growth in this region [3,4]. One approach to increase saline land use is to identify new plant species with salt tolerance [5,6]. However, a few plant species with low economic value can survive in coastal saline soils, including *Suaeda glauca* (Bunge) and *Tamarix chinensis* (Lour), because salt (NaCl) imposes several kinds of stress on plants [7,8]. Therefore, it is of great significance to introduce environmentally protective and economically valuable plants with salt tolerance in coastal saline areas. 

Vetiver grass [*Vetiveria zizanioides* (L.) Nash] is a perennial and herbaceous plant without seeds [9]. Because of its extensive and robust root system, vetiver grass has a superior advantage in adapting to a wide range of ambient stresses, including salinity, drought, flood, and heavy metals. In addition, vetiver grass is of high economic value because its fibrous roots contain essential oils, young leaves can be used as forage, and stems can be used as raw materials for papermaking [10,11]. Based on vetiver grass’s characteristics and economic value, it is recommended to introduce it into coastal saline soils for planting [10]. However, information on the feasibility of transferring vetivers into coastal saline regions is limited.

Salinity is a major environmental stressor. Salinity has detrimental effects on plant growth, development, and physiological and biochemical activities due to osmotic stress, specific ion toxicity, nutritional imbalance, or a combination of these factors [12,13]. Soil culture tests of saline irrigation showed that irrigation with <1% NaCl increased the biomass accumulation rate of vetiver, but this decreased at >1% NaCl [14]. Some studies confirm that the osmotic regulation of inorganic and organic osmolytes is the primary mechanism of vetiver tolerance to salt stress [15,16]. Hydroponic experiments with 100 mM NaCl (0.58% NaCl) for 9 days confirmed that inorganic ions contributed to osmotic adjustment in vetiver [*Vetiveria zizanioides* (L.) Nash] seedlings significantly (71.50–80.60%) compared with organic solutes (19.43–28.50%) [15]. Liu et al. [9] considered that the high ability of K^+^/Na^+^ for selective transportation might be the primary strategy for salt tolerance of vetiver grass due to the exclusion of Na^+^ from leaves or acceleration of K^+^ entering the leaf. Manea et al. [16] investigated the effect of varying salinity content on the nitrogen metabolism of vetivers. They observed that the nitrate-nitrogen content of the plant increased. However, little is known about the dose–effect relationship of salinity levels on osmotic regulators, nutrient uptake, growth, and feasibility of transplanting vetivers.

Therefore, the objectives of this study were to (1) investigate the dose–effect relationship of water salinity on root and shoot biomass, nutrient content (N, P, K, Na, Cl), malondialdehyde (MDA), osmotic regulators (reducing sugar and proline), and ion regulation in vetiver through pot experiment with different contents of NaCl, (2) analyze the salt tolerance of vetivers, and (3) exploit the feasibility of transferring vetivers into coastal saline regions. The study could provide a scientific basis and an essential reference for introducing vetivers in coastal areas.

## 2. Results

### 2.1. The Growth of Vetiver

**Figure 1:**
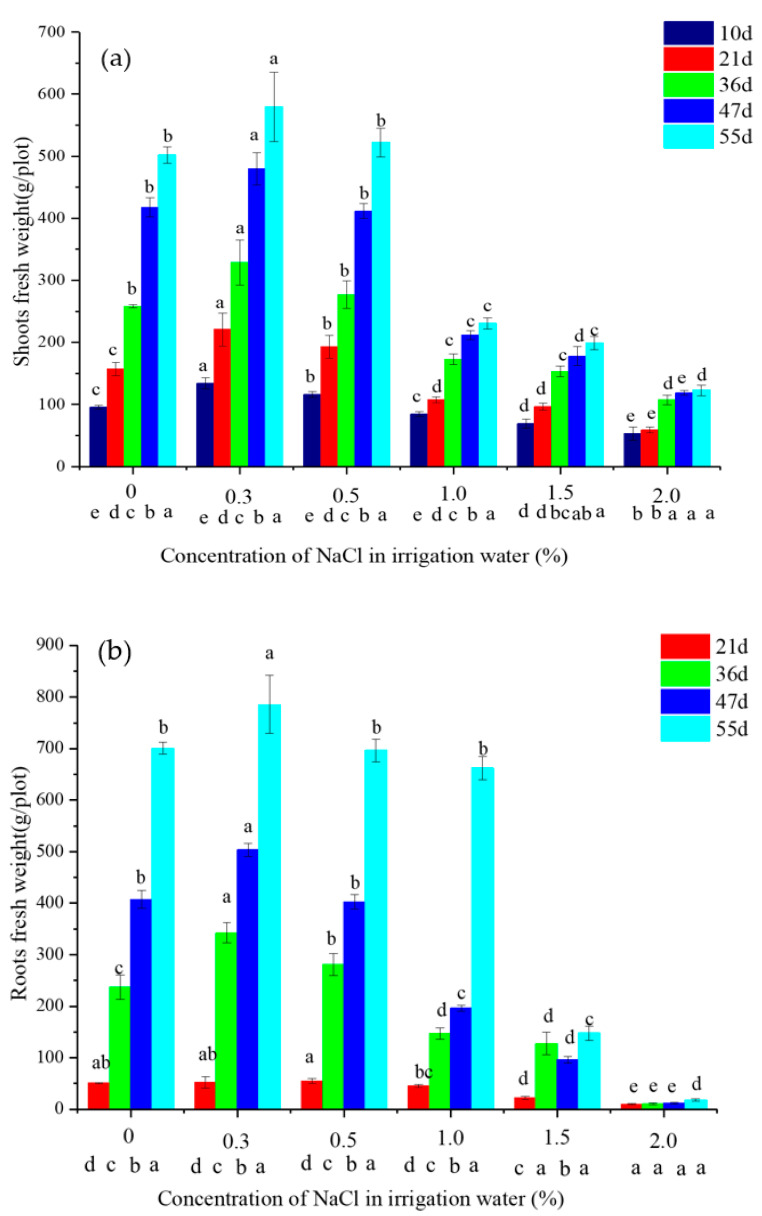
Fresh shoots (**a**) and roots (**b**) fresh weight of vetiver irrigated using water of varying NaCl content. Letters above the bars represent the significance of different salinities at the same time of growth (*p* < 0.05). Letters below the *x*-axis represent the significance of different growth times at the same salinity (*p* < 0.05). The data are shown as the mean ± standard deviation.

Significant differences in fresh weight of roots and shoots were determined with respect to NaCl content and time (Figure 1). The fresh weight of shoots and roots increased significantly with time at water salinities of 0, 0.3, 0.5, and 1.0%, but the differences were not always significant at 1.5 and 2.0% (Figure 1).

The fresh weight of shoots and roots irrigated with 0.3% saline water increased by 14.8–40.6% and 3.1–44.3%, respectively, compared with that of no saline water (CK). Fresh shoot and root weights of vetiver irrigated with 0.5% saline water significantly increased at 10, 21, and 36 d than CK, (Figure 1a), while no significant effect was observed after 47 d (Figure 1b). Compared with CK, the vetiver’s fresh shoot weight was significantly decreased by 11.7–54%, 27.8–60.3%, and 44.4–75.6% at water salinities of 1.0, 1.5, and 2.0%, respectively. The vetiver’s fresh root weight decreased by 5.5–51.8%, 46.3–78.9%, and 80.2–97.5% at water salinities of 1.0, 1.5, and 2.0%, respectively. Therefore, it could be concluded that the growth of the vetiver was significantly inhibited at >1.0% water salinity.

### 2.2. The Content of Nutrients in Vetiver

With increasing water salinity, the nitrogen (N) content in the roots and shoots of vetiver presented a wavy increasing trend. Minimum N content was observed at a salinity of 1.0% for both roots and shoots, while the maximum occurred at 1.5% for shoots and 0.5% for roots (Table 1). There were no differences in the N content of vetiver shoots compared with CK when grown in salinity levels of 0.3, 0.5, and 1.0%; N content significantly increased at 1.5 and 2.0% NaCl. The N content in the roots of vetiver increased by 24.6, 62.9, and 34.9% at 0.3, 0.5, and 2.0%, respectively, but decreased by 30.3 and 20.6% at 1.0 and 1.5%, respectively (Table 1). As for the phosphorus (P) content in shoots and roots, no significant difference was observed among the six water salinity levels (Table 1).

Compared with CK, the potassium (K) content in shoots increased by 34.3 and 18.5% at 0.3 and 1.0%, respectively, but no significant difference was observed among other salinity levels (Table 1). Compared with CK, the K content in the roots was significantly increased by 102.0, 98.0, 127.5, 89.8, and 91.5% at 0.3, 0.5, 1.0, 1.5, and 2.0%, respectively (Table 1). The sodium (Na) content in vetiver shoots decreased to the lowest value at 0.5% water salinity and subsequently increased until the end of the trial. Compared with CK, the Na content in the shoots decreased by 36.3 and 47.3% at 0.3 and 0.5%, but increased by 61.8, 227.9, and 172.7% at 1.0, 1.5, and 2.0%, respectively (Table 1). The Na content in the roots significantly increased with increasing water salinity (Table 1). Similarly, the chlorine content in the shoots presented a wavy upward trend when water salinity increased, with the maximum value observed at a water salinity of 2.0% (Table 1). Compared with CK, the chlorine (Cl) content in the roots increased by 148.4, 76.2, and 35.4% at 0.3, 0.5, and 2.0%, respectively, but decreased by 6.6 and 4.3 at 1.0% and 1.5%, respectively (Table 1). 

### 2.3. Malondialdehyde (MDA) Concentation

**Figure 2:**
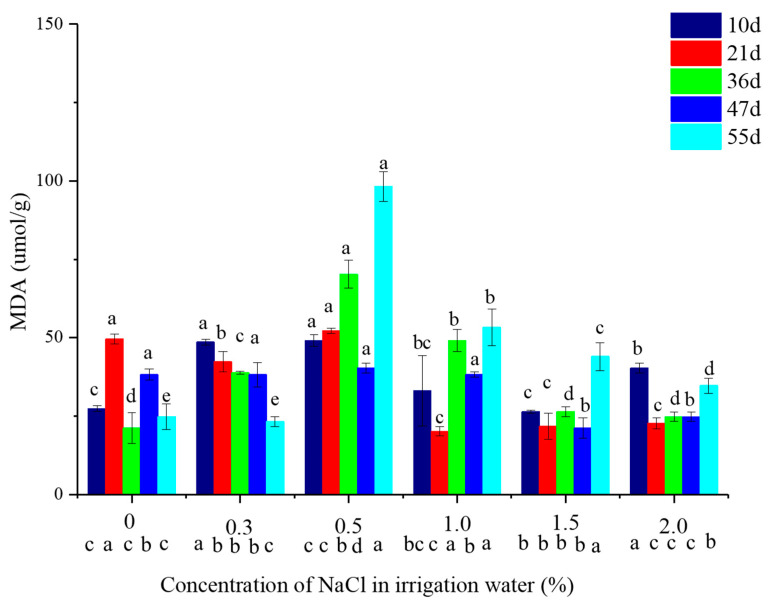
MDA concentration in shoots of vetiver at varying water salinities. Letters above the bars indicate the significance of different salinities at the same time of growth (*p* < 0.05). Letters below the *x*-axis indicate the significance of different growth times at the same salinity (*p* < 0.05). The data are shown as the mean ± standard deviation.

The MDA concentration of vetiver leaves irrigated with 0.3% saline water tended to decrease with time (Figure 2). The highest MDA concentration in these leaves (0.5–1.5% water salinity) was observed on day 55 (Figure 2). Generally, MDA concentration in leaves tended to increase first and then decrease with increasing water salinity during the trial period. The maximum of MDA in leaves was attributed to a water salinity of 0.5% (Figure 2). Compared with CK, no apparent differences were observed when irrigated with 0.3% saline water on day 55; However, the MDA concentration was greater by 295.8, 114.6, 77.1, and 39.6% with irrigation by 0.5, 1.0, 1.5, and 2.0% saline water on 55 d, respectively. 

### 2.4. The Content of Reducing Sugar and Prolin in Vetivere

**Figure 3:**
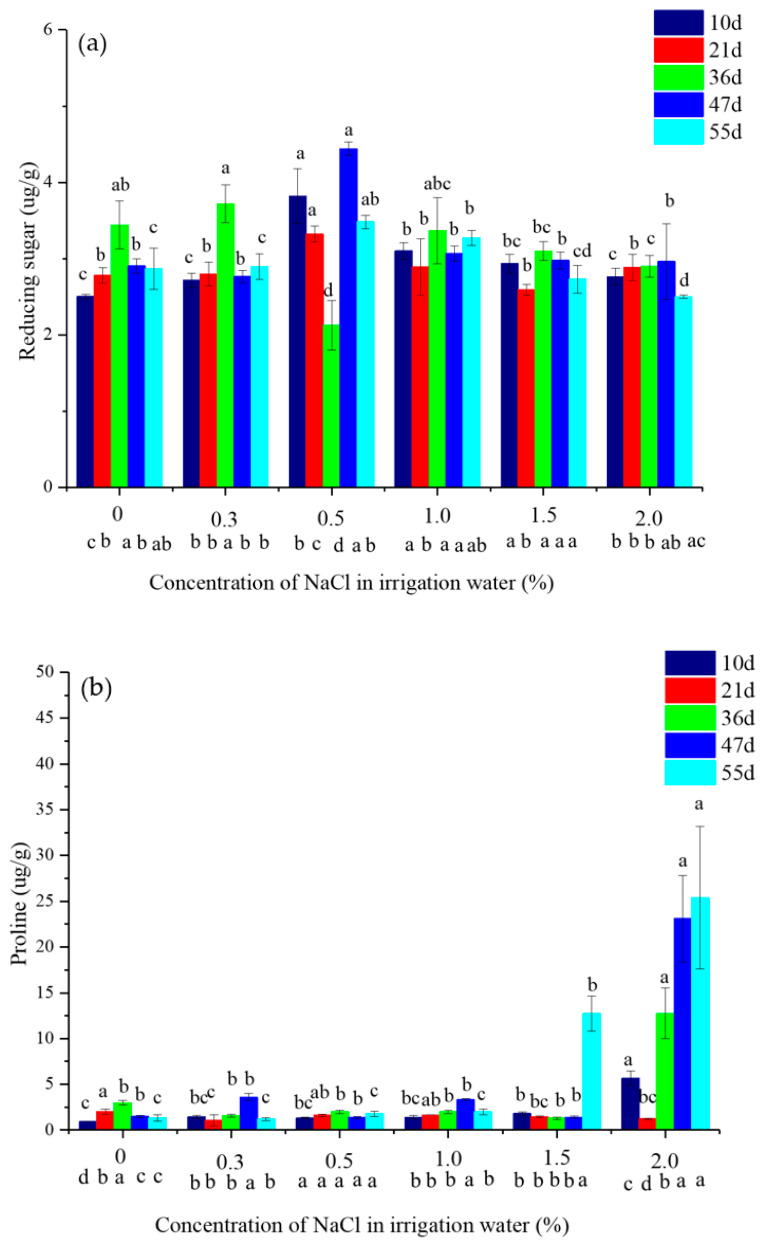
Reducing sugar (**a**) and proline (**b**) content in vetiver shoots at varying water salinities. Letters above the bars indicate the significance of different salinities at the same time of growth (*p* < 0.05). Letters below the *x*-axis indicate the significance of different growth times at the same salinity (*p* < 0.05). The data are shown as the mean ± standard deviation.

The apparent difference in reducing sugar and proline contents in vetiver leaves depended on water salinity and time (Figure 3). The content of reducing sugar in leaves of vetiver on day 10, 47, and 55 tended to increase first and then decrease with increasing salinity. At days 21 and 47, reducing sugar in leaves tended to increase and peak at a water salinity of 0.5%. No difference was observed at other water salinities (Figure 3a). Compared with CK, no apparent differences were observed with irrigation with 0.3 and 1.5% saline water at day 55; however, the content of reducing sugar in leaves of vetiver irrigated with 0.5 and 1.0% saline water was significantly greater by 21.5 and 14.2%, respectively. Leaves showed significantly lower reducing sugar (12.7%) when irrigated by 2.0% saline water at day 55. There was very low proline content in the leaves of vetiver irrigated with 0–1.0% saline water during the whole trial, excluding leaves irrigated with 2.0% saline water at day 21 (Figure 3b). Compared with CK, the proline content in leaves irrigated with 1.5 and 2.0% saline water significantly increased by 849.7 and 1793%, respectively, at day 55 (Figure 3b).

### 2.5. Ion Regulation in Vetiver

The transfer coefficients of potassium from roots to shoots were all >1 and ranged from 1.88 to 3.66. In contrast, the transfer coefficients of sodium from roots to shoots ranged from 0.18 to 0.91, which was <1 (Table 2). K tended to be concentrated in the shoots of vetiver, whereas Na tended to be concentrated in its roots. The transfer coefficients of Cl in vetivers irrigated with ≤0.5% saline water were lower than 1. However, the transfer coefficients of Cl in vetivers irrigated with ≥1.0% saline water were >1. This implies that Cl tends to be concentrated in vetiver roots under low NaCl stress and shoots under intense NaCl stress.

The ratios of K/Na (being > 1) in vetiver shoots tended to increase to a peak at a water salinity of 0.3%, and then decreased gradually until the water salinity reached 2.0% (Table 2). The K/Na ratios in vetiver roots tended to decrease with increasing water salinity (Table 2). These results suggest that the increasing water salinity may weaken the vetiver’s capacity for K uptake. The S_K/Na_ ratio increased at a water salinity of 0.3% and reached its highest at a salinity of 0.3–0.5%, subsequently decreasing until the salinity reached 2.0% (Table 2). These results indicate that low NaCl tolerance in vetiver may be attributed to Na^+^ exclusion from leaves or Na^+^ sequestration in roots, but high NaCl stress tolerance in vetiver may contribute to Na^+^ sequestration in roots.

## 3. Discussion

### 3.1. The Effect of Water Salinity Levels on Vetiver

Growth and physiological parameters can provide reliable criteria for evaluating salt stress or tolerance in plants [17]. In the present study, irrigation with low-salinity water promoted vetiver growth because fresh weights of vetiver roots and shoots were greater when irrigated with 0.3% saline water than CK (no saline water) (Figure 1). However, watering with >0.5% saline water began to reduce fresh shoot and root weight and inhibit growth. Significant inhibition was observed with >1.0% saline water, and the maximum saline tolerance was 2.0%. Therefore, it can be concluded that irrigation with 0.3% saline water promoted vetiver growth, but irrigation with 0.5, 1.0, and >1.5% saline water mildly, moderately, and strongly inhibited growth, respectively. In a pot experiment, it was observed that irrigation with <1% saline water increased the vetiver biomass [14]. Greenway et al. [18] reported that the application of <1.755% NaCl resulted in an apparent promotion of plant growth. Salinity can induce elemental nutrient deficiencies or imbalance in plants. In this study, low-salinity-induced vetiver growth may be due to salinity-induced acceleration of N, K, and Cl uptake by the vetiver. Due to the competition between nutrients and major salt species, the uptake and accumulation of nutrients by plants are often reduced under saline conditions [19]. The decrease in N content in roots might be related to the antagonistic relationship between toxic Cl^−^ and ${\mathrm{NO}}_{3}^{-}$ under 1.0 and 1.5% salinity [20]. However, the N content in roots was significantly increased due to an adaptation mechanism developed by the plants to overcome osmotic stress caused by salinity under 0.3 and 0.5% NaCl. Proline has also been considered a nitrogen source for growth and rapid recovery from stress in 2.0% saline water. Manea et al. [16] observed that irrigation of vetiver with 0.73–1.17% saline water increased nitrogen content in leaves compared with the control.

Under salinity stress, the plants induce the activity of antioxidative enzymes, including catalase, peroxidase, and superoxide dismutase, to defend against the increase in reactive oxygen species (ROS) that lead to lipid peroxidation in the cell membrane [21]. MDA is the main product of membrane lipid peroxidation in plants under salt stress, and its concentration represents the degree of cell membrane damage [21]. Irrigation with 0.3% saline water did not damage the membrane of the vetiver leaves. The MDA concentrations in the leaves irrigated with 0.3% saline water were similar to the control on day 55 (Figure 2). In addition, we found that antioxidant enzymes, including catalase, peroxidase, and superoxide dismutase, were significantly decreased in vetiver watered with 0.3% saline water in a previous study, which proved that irrigation with 0.3% saline water did not damage the cell membrane in vetiver leaves [22]. Irrigation with 0.5, 1.0, 1.5, and 2.0% saline water resulted in membrane damage in vetiver leaves, as seen by the significant increase of MDA in leaves compared to 0% NaCl in 55 d. In a previous study, MDA in leaves also showed a significant increase at 1.17% NaCl, but there were no significant changes at 0.56–0.88% NaCl, compared with the no NaCl treatment [9]. 

Osmotic regulators in plants are vital for sustenance under salinity stress, and these accumulate in plants to relieve environmental stress [23,24]. Plants synthesize several osmolytes to maintain osmotic balance [25]. Reducing sugars are well known as osmolytes and osmoprotectants. Proline is a nitrogenous compound that is a major osmolyte, part of defensive machinery that cope with salt stress. In the present study, no difference in proline and reducing sugars in leaves of vetiver was observed between 0.3% saline water and no saline water due to the vetiver’s growth promotion. The reducing sugars could contribute to the osmotic regulation in vetiver because reducing sugars were greater at irrigation with 0.5 and 1.0% saline water than with CK (Figure 3a). Vetivers enhance osmotic potential by accumulating reducing sugars to resist osmotic stress under salt stress [21,26]. The low content of proline in leaves irrigated with 0.5 and 1.0% saline water may be due to altered N metabolism [12,20], suggesting that proline did not play a significant role in osmotic adjustment under mild and moderate saline conditions. There was no significant difference in reducing sugar in leaves of vetiver irrigated with 1.5 and 2.0% saline water and CK, suggesting that reducing sugars do not significantly impact osmotic adjustment under strong saline conditions. The vetiver survival mechanism under high salt stress could be attributed to proline’s osmotic regulation because high proline levels were determined in leaves (Figure 3b). Therefore, sodium was accumulated in the roots and not easily transported to shoots, as seen by the low ratio of Na in shoots to roots (Table 2). In salt-tolerant and relatively salt-tolerant plants such as *Beta vulgaris* [27], *Brassica juncea* [28], Alfalfa [29], and sesame [30], sharp increases in proline levels were reported under salt stress. 

Various mechanisms have been reported in salt-tolerant plants that help control osmotic stress in cells. Tolerant plants reduce Na accumulation in the shoots by either reduced uptake, reduced root to shoot transport, compartmentalization of sodium into vacuoles, and/or salt extrusion from the surface [31,32]. In this study, the TCs of Na from roots and shoots irrigated with 0.3, 0.5, 1.0, 1.5, and 2.0% saline water were significantly lower than those without saline water, indicating that Na was accumulated in the roots and not transported to shoots. Plants minimize the harmful effects of ionic Na stress by excluding Na from leaf tissues and by compartmentalization of Na in roots [33,34]. Plants maintain high K^+^ and low Na^+^ contents to reduce the effects of NaCl under salt stress. A higher S_K_/_Na_ ratio indicates a more significant K/Na favoring K over Na accumulation in leaves [9,15]. In this study, the S_K_/_Na_ ratio was higher in all treatments than in CK, indicating that NaCl tolerance in vetiver may be attributed to Na^+^ sequestration in roots and Na^+^ exclusion in leaves. In addition, similar to Na^+^, Cl^−^ exclusion and Cl^−^ sequestration are essential for salt tolerance [35]. The TCs of Cl^−^ were lower than 1 in 0.3% and 0.5% saline water due to Cl^−^ sequestration in the vetiver’s roots. The TCs of Cl^−^ were greater than 1 in 1.0, 1.5, and 2.0% saline water, which may be because that the ability to transport Cl^−^ away from sensitive vetiver roots could be an important factor contributing to salt tolerance [36].

### 3.2. The Adaptability of Saline Soil to Transplanting and Introduction of Vetiver

The coastal saline soils of the Yellow River Delta, Laizhou Bay, southern shore of Bohai Bay cover an area of 1.24 million hectares. The area of mild (0.1–0.2% soil salinity), and moderate saline soils (0.2–0.4% soil salinity) accounted for 13.5% and 20.5%, respectively [37]. The introduction of vetivers in saline soil is recommended [10,38,39]. The soil salinity tolerance threshold for vetiver was reported to range from 8 dS/m (equivalent to 0.468% NaCl) to 31.8 dS/m (equivalent to 1.86% NaCl) [40,41]. According to shoot and root fresh weight and MDA, the optimal growth of vetiver occurred under irrigation with 0.3% saline water in the current study. Du et al. [38] stated that vetiver can be planted in saline soils under the influence of seawater with salinity from 8 mS/cm (equivalent to 0.468% NaCl) to 11 mS/cm (equivalent to 0.64% NaCl). The vetiver is ideal for growing in moderately saline soil (~0.3% salinity) under irrigation with low-salinity water (~0.3% saline water) since the present study confirmed the maximum of fresh plant weight of the vetiver (Figure 1). Therefore, vetiver is recommended for planting in slightly salinized soils (0.1–0.2% soil salinity), even moderately salinized soils (0.2–0.4% soil salinity) under irrigation with low-salinity water during transplanting in the coastal saline soils of the Yellow River Delta characterized by high Cl, Na, K [37].

The vetiver could be cultivated in highly salinized soils (0.4–0.6% soil salinity) under freshwater irrigation. In the present study, the vetiver tolerated 1.32% soil salinity (irrigation of 0.5% saline water) with normal tillering and growth (Figure 1 and Figure 2). The Yellow River water and desalinated seawater can be used as irrigation water during the transplanting period. The pot experiments were carried out to further verify the adaptability of the vetiver in coastal saline soil and its irrigation management.

## 4. Materials and Methods

### 4.1. The Experimental Design

Seedlings of vetiver grass [*Vetiveria zizanioides* (L.) Nash] were obtained from the Jiangxi Academy of Forestry, Jiangxi Province. The seedlings (4 strains/pot) were uniformly cut to a height of approximately 14 cm (required for their growth) and transplanted into 90 pots (15 cm in diameter and 25 cm in height) pre-filled with 15 kg of soil/pot (pH 6.9, total salinity of 0.32%, total K of 4.4 g·kg^−1^, total N of 18.2 g·kg^−1^, total P of 2.08 g·kg^−1^) to establish and grow under normal conditions with proper irrigation for 14 days. Thereafter, the plants were watered with approximately 1-L/pot of increasing concentrations of NaCl, i.e., 0 (CK), 0.3, 0.5, 1.0, 1.5, and 2.0% every two days from 5 August to 27 September. The cumulative soil salt was analyzed by multiplying the content of NaCl by the volume and time of watering, then dividing the quality of the soil, reaching approximately 0.32 (CK), 0.85, 1.22, 2.12, 3.02, and 3.92% after 55 days based on the estimation of the amount of irrigated salt. There were 15 pots for each salt level, and three pots (triplicates) were sampled at each sampling during the trial.

### 4.2. Determination of Plant Samples

Vetiver grass samples were collected at 10, 21, 36, 47, and 55 d. At sampling, the plant height and tiller number were recorded, and the vetiver from each treatment was divided into two parts (shoots and roots) and then rinsed with tap water and deionized water. Plants were air-dried on absorbent paper to determine the fresh weight of the shoots and roots. Fresh plant samples were used to determine malondialdehyde (MDA), reducing sugar, and proline; other plant samples were dried in an oven at 100 °C for the determination of N, P, K, Na, and Cl.

The MDA concentration in 1 g of fresh leaves was determined by the thiobarbituric acid method [42]. Two milliliters of supernatant was combined with 2 mL of 0.75% thiobarbituric acid, heated in boiling water for 15 min, and cooled rapidly on ice. The mixture was then centrifuged in 4000 rpm, and the absorbance was measured at 535 nm using an extinction coefficient of 156 mM^−1^ cm^−1^. The free proline content in fresh leaves (0.5 g) was determined by the ninhydrin method [43]. Fresh leaves were homogenized in 3% (*w*/*v*) sulfosalicylic acid and then centrifuged. The mixture was heated at 96 °C for 1 h in a water bath after the addition of acid ninhydrin and glacial acetic acid. The reaction was then stopped using an ice bath. The mixture was extracted with toluene, and the absorbance of the fraction with toluene aspired from the liquid phase was read at 520 nm. The method detected proline in the 0.5 to 35.0 μg/g range of fresh weight leaf material. The content of reducing sugar in fresh leaves (0.5 g) was determined by the dinitrosalicylic acid method [44]. Plant and soil samples were digested with H_2_SO_4_-H_2_O_2_ to determine total nitrogen using the Kjeldahl method, total P by molybdenum blue, total K, and Na by flame photometry, and total Cl by silver nitrate titration [45]. Soils were extracted with H_2_O to determine total soil salt content using the mass method [45].

The transfer coefficient of nutrients (ratio of the element in shoots to the corresponding elements in roots) was used to assay nutrient transportation from the roots to the shoots. The ability of K and Na ion-selective transportation (SK/Na) was also calculated using Equation (1) [9,15].

(1) $$S_{K/Na}=\frac{K/Na\;in\;shoots}{K/Na\;in\;roots}$$

### 4.3. Data Statistical Analysis

Graphical analysis was carried out using Origin Pro 9.0 (OriginLab Corporation, Northampton, MA, USA). Statistical analyses (one-way analysis of variance) were performed using SPSS Version 19.0 software (SPSS Inc., Chicago, IL, USA). The least significant difference (LSD) was used to test for significance at *p* < 0.05.

## 5. Conclusions

The present results indicate that irrigation with saline water (≤0.3% NaCl) promoted vetiver’s growth during the transplanting period due to NaCl-induced promotion of N and K uptake, salt stress adaptation, and Na^+^ sequestration and Cl^−^ sequestration in roots. Significant inhibition was observed at 0.5, 1.0, 1.5, and 2.0% saline water. The inhibition of vetiver growth by high water salinity levels was attributed to NaCl stress-induced cell membrane damage in shoots based on MDA concentration. The transport of Cl^−^ away from sensitive roots, Na^+^ sequestration in roots, Na^+^ exclusion in leaves, and organic osmolytes (reducing sugar, proline) driven osmotic regulation are functions of vetiver resistance to salt.

Therefore, vetiver is recommended for planting in slightly salinized soils (0.1–0.2% soil salinity) and even moderately salinized soils (0.2–0.4% soil salinity) under irrigation with low-salinity water during transplanting. The vetiver could be cultivated in highly salinized soils (0.4–0.6% soil salinity) under freshwater irrigation.

## Figures and Tables

**Table 1:** Contents of N, P, K, Na (g/kg) and Cl (mg/kg) in vetiver plants collected 55 d after irrigation with saline water.

		0 (CK)	0.3	0.5	1	1.5	2
Shoots	N	3.7 ± 0.7b	4.4 ± 0.7b	4.2 ± 1.3ab	2.6 ± 0.5b	6.5 ± 1.2a	6.0 ± 1.0a
	P	1.5 ± 0.2a	1.5 ± 1.0a	1.5 ± 0.1a	1.5 ± 0.3a	1.8 ± 0.2a	1.5 ± 0.5a
	K	10.8 ± 1.9b	14.5 ± 0.8a	11.0 ± 1.1bc	12.7 ± 0.5b	10.5 ± 0.6b	11.0 ± 0.9b
	Na	1.65 ± 0.26d	1.05 ± 0.06d	0.87 ± 0.02e	2.67 ± 0.29c	5.41 ± 0.18a	4.50 ± 0.25b
	Cl	3.34 ± 0.92c	4.83 ± 0.43b	3.67 ± 0.049c	7.34 ± 1.83ab	5.70 ± 1.20b	8.57 ± 1.41a
Roots	N	17.5 ± 1.1c	21.8 ± 1.0b	28.5 ± 0.8a	12.2 ± 1.8e	13.9 ± 1.5d	23.6 ± 0.8b
	P	0.88 ± 0.17a	0.87 ± 0.10a	0.89 ± 0.17a	0.90 ± 0.26a	0.64 ± 0.26a	0.89 ± 0.35a
	K	2.95 ± 0.16c	5.96 ± 0.15b	5.84 ± 0.18b	6.71 ± 0.15a	5.60 ± 0.20b	5.65 ± 0.37b
	Na	1.82 ± 0.15e	5.03 ± 0.59d	4.85 ± 0.29d	10.56 ± 1.27c	18.91 ± 1.01a	17.40 ± 0.66b
	Cl	3.95 ± 0.15c	9.81 ± 1.30a	6.96 ± 1.45b	3.69 ± 0.65c	3.78 ± 0.22c	5.35 ± 0.64b

Note: Values followed by different letters within a row indicate significance at *p* < 0.05 (LSD) for NaCl solution levels.

**Table 2:** Transfer coefficients (TCs) of K, Na and Cl, and ratios of in shoots (R_shoot_) and roots (R_root_), and S_K/Na_ in plants of vetiver collected 55 d after watering with saline water.

		0 (CK)	0.3	0.5	1	1.5	2
	K	3.66	2.43	1.88	1.89	1.88	1.95
TCs	Na	0.91	0.21	0.18	0.25	0.29	0.26
	Cl	0.85	0.49	0.53	1.99	1.51	1.6
R_shoot_	K/Na	6.54	13.81	12.64	4.76	1.94	2.44
R_root_	K/Na	1.62	1.19	1.2	0.64	0.3	0.33
S_K/Na_	K/Na	4.04	11.61	10.53	7.44	6.47	7.39

Note: S_K/Na_ = (K/Na in shoots)/(K/Na in roots).

## Data Availability

Not applicable.
